# Foot Pronation Prediction with Inertial Sensors during Running: A Preliminary Application of Data-Driven Approaches

**DOI:** 10.5114/jhk/163059

**Published:** 2023-07-15

**Authors:** Liangliang Xiang, Yaodong Gu, Alan Wang, Vickie Shim, Zixiang Gao, Justin Fernandez

**Affiliations:** 1 Faculty of Sports Science, Ningbo University, Ningbo, China.; 2 Auckland Bioengineering Institute, The University of Auckland, Auckland, New Zealand.; 3 Faculty of Medical and Health Sciences, The University of Auckland, Auckland, New Zealand.; 4 Faculty of Engineering, University of Pannonia, Veszprém, Hungary.; 5 Department of Engineering Science, Faculty of Engineering, The University of Auckland, Auckland, New Zealand.

**Keywords:** foot pronation, running, inertial measurement sensors (IMU), machine learning, one-dimensional convolutional neural networks (CNN1D)

## Abstract

Abnormal foot postures may affect foot movement and joint loading during locomotion. Investigating foot posture alternation during running could contribute to injury prevention and foot mechanism study. This study aimed to develop feature-based and deep learning algorithms to predict foot pronation during prolonged running. Thirty-two recreational runners have been recruited for this study. Nine-axial inertial sensors were attached to the right dorsum of the foot and the vertical axis of the distal anteromedial tibia. This study employed feature-based machine learning algorithms, including support vector machine (SVM), extreme gradient boosting (XGBoost), random forest, and deep learning, i.e., one-dimensional convolutional neural networks (CNN1D), to predict foot pronation. A custom nested k-fold cross-validation was designed for hyper-parameter tuning and validating the model’s performance. The XGBoot classifier achieved the best accuracy using acceleration and angular velocity data from the foot dorsum as input. Accuracy and the area under curve (AUC) were 74.7 ± 5.2% and 0.82 ± 0.07 for the subject-independent model and 98 ± 0.4% and 0.99 ± 0 for the record-wise method. The test accuracy of the CNN1D model with sensor data at the foot dorsum was 74 ± 3.8% for the subject-wise approach with an AUC of 0.8 ± 0.05. This study found that these algorithms, specifically for the CNN1D and XGBoost model with inertial sensor data collected from the foot dorsum, could be implemented into wearable devices, such as a smartwatch, for monitoring a runner’s foot pronation during long-distance running. It has the potential for running shoe matching and reducing or preventing foot posture-induced injuries.

## Introduction

Although running is one of the most common forms of exercise and provides numerous benefits for health, runners suffer from a high prevalence of running-related injuries, especially in the lower limbs (range: 18.2 to 92.4%) (Dempster et al., 2021; Saragiotto et al., 2014). Abnormal foot postures may affect foot movement and joint loading during locomotion. It is also associated with an increased risk of lower extremity injuries, such as medial tibial stress syndrome and patellofemoral pain (Neal et al., 2015). Foot posture may change during long-distance running (Mei et al., 2019). Investigating foot posture alterations during running could contribute to understanding foot injury mechanisms and injury prevention.

The foot tends to be more pronated at the middle and the end phase of distance running (Dos Santos et al., 2019). Foot pronation refers to the foot being inwardly rotated in its subtalar joint axis and this usually occurs during the first 40–50% of foot contact during locomotion (Behling et al., 2020; Nigg et al., 2019). Foot pronation during running contributes to absorbing foot-ground contact loading. However, over-pronation could change the lower limb joint coordinate and moment, and increase injury risk (Malisoux et al., 2016). Dos Santos et al. (2019) found that an increased foot pronation angle is associated with increased running speed. Furthermore, foot pronation is widely discussed with regard to footwear development (Behling et al., 2020; Pan et al., 2023). Malisoux et al. (2016) illustrated that recreational runners might benefit from motion control shoes, especially those with pronated feet, compared to standard running shoes. However, changes in the subtalar axis are difficult to assess during running (Behling et al., 2020). Therefore, evaluating foot pronation during running in real-time is quite challenging.

Machine learning algorithms have been widely implemented in the gait biomechanics realm over the past decades (Halilaj et al., 2018; Xiang et al., 2022a). Principle component analysis (PCA) is a commonly used dimensionality reduction technique for biomechanics data to avoid the curse of dimensionality (Kobsar et al., 2014). Support vector machine (SVM) was utilized to detect age-related differences from gait biomechanics data. Runners’ levels of experience could be classified from their lower limb kinematic and kinetic data (Clermont et al., 2019) using SVM. It can also be applied in injury rehabilitation monitoring. Random forest (RF) algorithms can classify the inclination conditions of running surfaces using a single inertial wearable sensor (Ahamed et al., 2019). Gradient boosted (GB) decision tree and regression tree were utilized to predict ground reaction force (GRF) and running surface identification (Dixon et al., 2019). Feature engineering plays a crucial role in traditional machine learning as it is the process of selecting, manipulating, and transforming the raw data into desired features that will be used as the inputs into model training. A model’s performance may be directly affected by the feature engineering.

Deep learning algorithms integrated with inertial sensors have emerged in recent years, as they are convenient and can capture biomechanical data outside the traditional laboratory (Halilaj et al., 2018; Hernandez et al., 2021). The acceleration of the shank and foot (Ngoh et al., 2018) feeding the artificial neural networks (ANN) could predict GRF during running. Human activity could be accurately recognized by inertial measurement unit (IMU) sensors using one-dimensional convolutional neural networks (CNN1D) and long-short term memory (LSTM) networks (Ordóñez and Roggen, 2016). Due to time-series having a strong one-dimensional (time) locality, it has powerful information extraction capabilities, and convolution kernels can be trained as the templates to make predictions (Dixon et al., 2019; Dorschky et al., 2020; Tan et al., 2020). A previous study found that lower limb joint angles and moments are predictable using CNN1D (Dorschky et al., 2020). CNN1D were also adopted to calculate and estimate spatiotemporal gait parameters and showed high accuracy (Zrenner et al., 2018). Tan et al. (2020) estimated the vertical average loading rate (VALR) during running via CNN1D. It could also accurately detect outdoor terrain types using the accelerometry data measured from the tibia (i.e., concrete, synthetic, and wood chip surfaces) (Dixon et al., 2019). Due to the small sample size in running biomechanics, cross-validation approaches, such as leave-one subject out, have emerged to be used for model optimization and training in the biomechanical field (Hernandez et al., 2021).

However, no study has monitored the foot posture of runners using wearables and machine learning algorithms. Classifying foot posture of the running gait deserves attention to benefit the running shoe industry and provide running recommendations for runners. Furthermore, it could contribute to injury prevention for runners prone to pronation. Therefore, the objective of this study was to develop feature-based and signal-based models to predict foot pronation during distance running. It was hypothesized that (1) machine learning could reliably predict foot pronation during running and that deep learning would overperform the SVM and tree-based models on foot pronation; and (2) adding synthetic data to the training dataset using time-series data augmentation methods could enhance model robustness and improve prediction accuracy.

## Methods

### Participants

According to a previous evidence-based study (Xiang et al., 2022a), a sample size of no less than 20 should be appropriate to conduct this study. Thirty-two recreational runners (age: 25.8 ± 3.0 yrs; body height: 1.77 ± 0.06 m; body mass: 78.2 ± 4.9 kg; BMI: 24.9 ± 1.7 kg/m^2^) were recruited for this study from universities and local running clubs via posters and social media. All of them met the criteria of a minimum running volume of 20 km/week and were free from neural disorders and without any lower limb musculoskeletal injuries in the past six months. Subjects with an abnormal foot shape or posture, such as pes cavus, were excluded. Participants were informed of the test procedure, requirements, and the study objective. They were free to leave the study at any moment without giving a reason, and written informed consent was obtained before the test.

### Experimental Protocol and Data Collection

**Figure 1:**
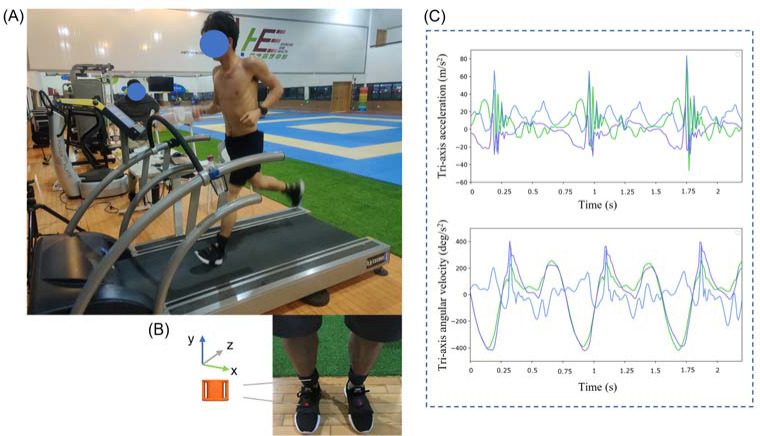
A. Pictorial illustration of the experimental setting; B. Display of the coordinate system and locations of IMU sensor placements; and C. Graphical representation from the inertial sensor showing the tri-axis acceleration and angular velocity output.

Each participant’s foot posture was documented using the foot posture index-6 (FPI-6) scale (Redmond et al., 2006). Only participants with feet categorized as neutral feet pre-running were included in this study. Participants were allowed to become familiar with the experimental environment within ten minutes. Each runner was provided with the same neutral running shoes (heel height: 33 mm, EVA midsole). In this study, 9-axial IMU sensors (IMeasureU, Auckland, New Zealand; Mass: 12 g; Range: ±16 g (accelerometer), ± 2000 °/s (gyroscope), and ± 1200 μT (magnetometer); Resolution: 16 bit; Sample rate: 100 Hz) were attached to the right dorsum of the foot and the vertical axis of the distal anteromedial tibia (3 cm away from the crest of the medial malleolus) using bandages and straps (Figure 1).

Runners ran on a treadmill (Quasar, h/p Cosmos®, GmbH, Germany) at a pace of 11.2 ± 1.2 km/h. The modified Borg Rating of Perceived Exertion (RPE) was used to control the running intensity during running. FPI-6 was assessed before and after 10 km of running while standing on the smooth floor with their feet shoulder-width apart. Eighty seconds of linear acceleration and angular velocity from inertial sensors were recorded for each runner at the beginning and after 10 km of running. The data were gathered in the IMeasureU Research (IMeasureU, Auckland, New Zealand) via Bluetooth connection with an iPad 2018 (Apple Inc., California, USA). A single experienced practitioner conducted all pre- and post-running foot posture evaluations.

FPI-6 has good clinometric validation and reliability and is a widely used foot posture tool in experimental (Ryan et al., 2014) and clinical (Redmond et al., 2008) investigations. Foot posture was evaluated as neutral foot (0 < FPI-6 < 6), foot pronation (FPI-6 score > 6), and foot supination (0 < FPI-6 < 0) according to Redmond et al. (2006). The foot pronated after running for twenty-eight runners, while four runners maintained a neutral posture. To avoid the data imbalance issue for the classification task, only data from twenty-eight runners was further used in machine learning training. For each trial of acceleration and angular velocity data, the first and last 10 s were excluded to reduce running transition effects (Dixon et al., 2019).

### Machine and Deep Learning

**Figure 2:**
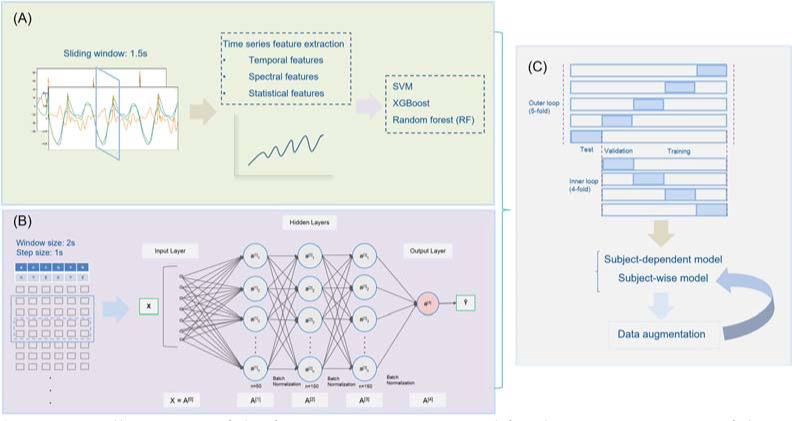
A. Illustration of the feature engineering used for data preprocessing of three feature-based machine learning algorithms; B. Convolutional neural networks; and C. Nested k-fold cross-validation structure for hyper-parameter tuning and data augmentation.


This study explored classical machine learning algorithms, including SVM, extreme gradient boosting (XGBoost) and RF, to predict foot pronation (Figure 2). CNN1D was employed as the deep learning approach. Each model was built with three input conditions (i.e., tibia sensor, dorsum sensor, and tibia + dorsum sensor). Accuracy, precision, recall, F1-score, Matthews correlation coefficient (MCC), and the area under the receiver operating characteristic (ROC) curve (AUC) metrics were employed to evaluate the classifier’s performance.

$$\mathrm{Pr}ecision=\frac{True\;Positive}{True\text{ Positive+False Positive}}\;\left(1\right)$$

Recall=True PostiveTrue Positive+False negative 2

F1−score=2*Precision*RecallPrecision+Recall 3

### Feature-Based Machine Learning

TSFEL library (Barandas et al., 2020) was used to extract features of acceleration and angular velocity from statistical (e.g., autocorrelation), temporal (e.g., variance), and spectral (e.g., mean coefficient of Fast Fourier Transform) domains. A window size of 1.5 s was adopted to extract features to cover at least one gait cycle during each iteration. A 2184 × 1260 matrix was extracted from the tibia sensor/dorsum sensor, including 108, 216, and 936 features from the temporal, statistical, and spectral domains, respectively. Feature selection was further implemented for dimensionality reduction. First, a filter was applied to remove highly correlated features with a threshold of 0.95. Second, low variance features were discarded with a set threshold of 0.1. Finally, 757, 765, and 1522 features were retained for the tibia sensor, dorsum sensor, and tibia plus dorsum sensors, respectively.

### Deep Learning

In the CNN1D model, each feature was standardized (*S_i_*) to a mean (*µ*) of 0 and a standard deviation (σ) of 1 before feeding into the deep learning model (Formula 4) (Kettaneh et al., 2005).


$$S_{i}=\frac{x_{i}-\mu }{\sigma }\;\left(4\right)$$

A window size (*W*) of 200 and a step size of 100 were adopted to feed input data to train the model. Therefore, the input of this CNN1D model would be concatenated as 2D matrices: *X*=R ∈ *R*^W*(6*n)^. The activation function of hidden layers was defined as a rectified linear units (ReLU, Formula 5) function because it could significantly accelerate the convergence of stochastic gradient descent and avoid the vanishing gradient problem during training compared to other activation functions such as sigmoid and hyperbolic tangent (Tanh) (Dixon et al., 2019). The activation function in the output layer was adopted as a sigmoid function (Formula 6).


$$f\left(x\right)=\frac{1}{1+e^{-x}}\;\left(5\right)$$

$$f\left(x\right)=\left\{\begin{array}{c} 0,\;if\;x<0\\ x,\;if\;x\geq 0 \end{array}\;\left(6\right)\right.$$

The adaptive Moment Estimation (Adam) optimizer was used for gradient descent optimization with a learning rate of 10^-3^. A binary cross-entropy function was applied as the loss function, and the model was evaluated by accuracy metrics. A batch normalization technique was used to reduce the training parameters and avoid overfitting. Each model was trained for 20 epochs with a batch size of 100. The CNN1D was built in the Keras framework with Tensorflow as the low-level API backend.

### Nested k-Fold Cross-Validation

Three input conditions were investigated for each model, incorporating tibia sensor, dorsum sensor, and combined tibia and dorsum sensors data. The model’s performance of subject-wise (data from one participant only included in one dataset (training, validation, or testing)) and record-wise (randomly split data into training, validation, and test datasets) approaches was cross-validated. A custom nested k-fold cross- validation was designed to validate the model’s performance, comprising a four-fold inner loop and a five-fold outer loop (Hernandez et al., 2021). An inner loop was used for hyper-parameter tuning and validation; the test dataset was included using an outer loop. Hence, this study separated the ratio of train, validation, and test datasets into 60%, 20%, and 20%.

### Hyper-Parameters Selection

As shown in Table 1, the hyperparameters of the number of estimators, max depth, and criterion were tuned for the RF algorithm. The number of estimators, max depth, and learning rate were tuned for the XGBoost algorithm. The kernels, C, and gamma were tuned in the support vector classifier (SVC). The hyperparameters of CNN1D included the number of layers (1, 2, 3), neurons (50, 100, 150, 200), kernel (10, 15, 20), and batch size (50, 100, 200). There were three hidden layers with 50 neurons for the first layer and 150 neurons for the second and third layers. Each CNN1D layer was filtered with a kernel size of 15.

**Table 1:** Parameter selections for SVM, XGBoost, and RF classifiers. Parameters in the record-wise model are represented on the left side of ‘/’, and the right side represents the subject-independent model.

	SVM			XGBoost			RF		
Parameters	Types of kernels	C	gamma	The number of estimators	Max depth	Learning rate	The number of estimators	Max depth	Learning rate
Candidates	linear /rbf	0.001, 0.01, 0.1	0.001, 0.01, 0.1	from 20 to 40 with increment of 5	from 10 to 20 with increment of 2	1, 1.2, 1.4, 1.6	50,100, 200,400	10,20,40	entropy, gini
Tibia	linear	0.01	0.001	35	16	1/1.2	100/400	20/40	entropy/gini
dorsum	linear	0.01/0.001	0.001	35/40	18/12	1/1.2	200/400	40	gini
Tibia & dorsum	linear	0.01/0.1	0.001	20/30	12/16	1.4/1	100/400	40	gini/entropy

Note: SVM: support vector machine; RF: random forest; XGBoost: extreme gradient boosting

### Data Augmentation

Data augmentation leverages limited data by transforming the original samples to create new ones (Um et al., 2017). Several time-series data augmentation approaches were used to improve the training dataset from the foot dorsum for the subject-independent model to enhance the CNN1D model’s generalization and demonstrate whether the model’s performance could be further improved with a greater sample size. Specifically, these approaches included adding noise, time scale, and time warp to the time-series data. As a result, training data was twice as large as during model training. Additionally, we tripled the training data by combining adding noise with the time scale and time warp. Data jittering was done by adding Gaussian noise with a mean *µ* = 0 and a standard deviation *σ* = 0.03 to the time series. The time scale was determined by scaling time series with random scalars from a Gaussian distribution with *µ* = 1 and *σ* = 0.1. Time warping was accomplished by warping the time series with a smooth cubic spline-based curve consisting of four knots, and each knot had a random magnitude from a Gaussian distribution with *µ* = 1 and *σ* = 0.2.

## Results

### Machine Learning Approaches

**Figure 3:**
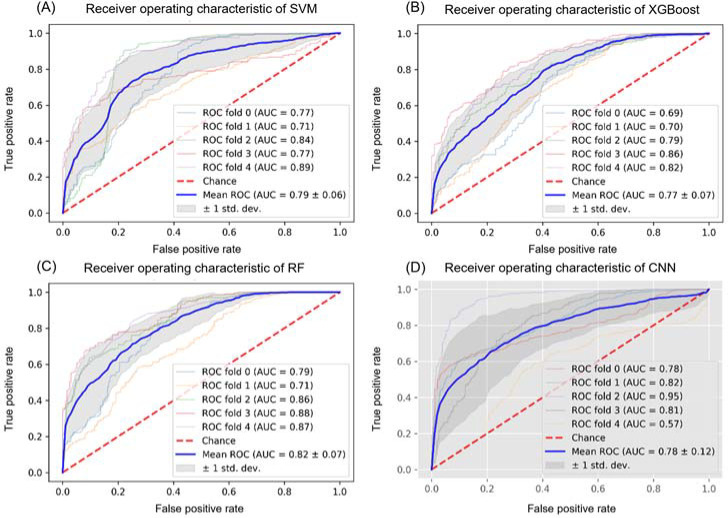
A. B. and C. ROC curve and AUC of the feature-based machine learning approaches on the tibia and dorsum of the foot; and D. ROC curve and AUC of CNN1D on the tibia and dorsum of the foot. Note: SVM: support vector machine; RF: random forest; XGBoost: extreme gradient boosting; CNN1D: one-dimensional convolutional neural networks; ROC: receiver operating characteristic; AUC: the area under ROC curve

The SVM classifier achieved the best accuracy among three feature-based approaches using the input of acceleration and angular velocity data collected at the distal tibia and foot dorsum. Accuracy and AUC were 74.7 ± 6.9% and 0.79 ± 0.06 for the subject-independent and 97.3 ± 0.4% and 0.99 ± 0 for the record-wise methods (Table 2 and Figure 3). The best accuracy in the XGBoost classifier was 74.7 ± 5.2% for the subject-wise and 98.0 ± 0.4% for the record-wise methods based on the dataset of the foot dorsum. The best accuracy in the RF was shown in the two-sensor model (71.3 ± 5.3% and 98.8 ± 0.3%), with AUC = 0.82 ± 0.07 and 0.99 ± 0. A detailed model performance analysis for SVM, XGBoost, and RF is presented in Table 2.

**Table 2:** Accuracy, Precision, Recall, F1 score, and MCC of subject independent and dependent models. Values in the record-wise model are represented on the left side of the ‘/’, and the right side represents the subject-independent model.

		Precision	Recall	F1 score	Accuracy	MCC
		SVM				
Tibia	Neutral	74.1(6.4)/85.3(12)	56.1(21.3)/76.1(25.1)	60.8(11.4)/78.5(19.5)	66.9(4.1)/ 96.3(0.7)	37.1(7.6)/ 92.5(1.5)
	Pronate	66.7(10.8)/81.4(16.6)	78(13.2)/87.2(13.1)	70(1.5)/83.1(13.2)		
Dorsum	Neutral	74.1(3.5)/85.6(11.8)	68(8.8)/82.3(15.7)	70.5(5.1)/83.7(13.7)	71.7(3.5)/ 96.9(0.4)	44(6.7)/ 93.8(0.7)
	Pronate	70.3(5.2)/83.5(13.7)	75.7(5)/86.3(11.3)	72.6(2.6)/84.7(12.3)		
Tibia & Dorsum	Neutral	72.5(7.5)/85.1(13.7)	81.2(7.7)/89(9.6)	76.3(6.1)/86.8(11.3)	**74.7(6.9)**/ **97.3(0.4)**	50.3(13.7)/ 94.5(0.8)
	Pronate	78.7(7.9)/87.8(10.7)	68.3(11.5)/83(16.8)	72.6(8.5)/84.9(13.7)		
		XGBoost				
Tibia	Neutral	68.3(9.9)/82.9(16.2)	60.2(7.7)/78.5(19.1)	63.5(6.2)/80.3(17.3)	65.4(6.4)/ 97.1(0.6)	31.5(13.7)/ 94.2(1.2)
	Pronate	63.9(6.2)/80.3(17)	70.6(11)/84.1(15.5)	66.8(6.9)/81.9(16)		
Dorsum	Neutral	76.4(5.6)/87.1(11.5)	73(11.3)/85.6(14.9)	74.1(6.3)/86.1(12.8)	**74.7(5.2)/** **98(0.4)**	50.2(10.6)/ 96(0.9)
	Pronate	74.5(8.5)/86.3(13.2)	76.6(7.3)/87.2(11.8)	75(4.7)/86.5(11.9)		
Tibia & Dorsum	Neutral	65.3(4.1)/81.4(16.4)	71.6(17)/84.7(17.7)	67.5(9.8)/82.5(16.6)	67(6.1)/ 97.6(0.6)	35.2(12.7)/ 95.2(1.3)
	Pronate	71.3(10.3)/84.5(15.1)	62.4(8.6)/80(18.6)	65.4(4.7)/81.5(16.4)		
		RF				
Tibia	Neutral	69.7(17)/84.2(18.8)	54.6(10.7)/75.9(22.6)	59.9(8.2)/78.9(19.9)	63.2(9.4)/ 98(0.4)	28.4(21)/ 95.9(0.7)
	Pronate	60.9(9)/79.1(19.2)	71.8(17.5)/85.2(18.3)	65.3(10.8)/81.6(18)		
Dorsum	Neutral	77.2(16.4)/87.9(15.8)	60.1(16.1)/79.5(22.5)	65.8(11)/82.3(18.2)	69.4(9.1)/ 98.7(0.4)	41.6(20)/ 97.5(0.9)
	Pronate	67.3(10.5)/83.1(17.5)	79(14.9)/88.8(14.4)	71.7(8.3)/85.2(14.7)		
Tibia & Dorsum	Neutral	68.9(6.1)/83.6(15.3)	79.8(12.2)/89.6(13.1)	73.3(5.6)/86(13.4)	**71.3(5.3)**/ **98.8(0.3)**	44.6(11)/ 97.5(0.6)
	Pronate	78(11.2)/88.7(13.3)	62.7(13.1)/80.4(20)	68(7.5)/83.4(16.3)		
		CNN1D				
Tibia	Neutral	73.3(12.5)/96.2(1.8)	58.4(18.4)/90.5(2.5)	63.2(13.6)/93.3(1.2)	68.3(9.8)/ 93.6(1)	38.1(19.6)/ 87.4(1.8)
	Pronate	67.2(10.6)/91.3(2.5)	77.9(11.1)/96.7(1.4)	71.5(8.3)/93.9(0.9)		
Dorsum	Neutral	81.6(2.7)/97.2(0.6)	61.6(13.1)/88.8(1.6)	69.3(6.7)/92.8(0.8)	**74(3.8)**/ 93.2(0.6)	50(7)/ 86.7(1.1)
	Pronate	70.9(7.6)/90(1.1)	86.1(5.5)/97.5(0.6)	77.2(1.8)/93.6(0.6)		
Tibia & Dorsum	Neutral	76.6(8.8)/96.1(1.2)	66.8(16.3)/92.6(2.1)	70.8(11.9)/94.3(1.1)	73.9(9.5)/ **94.5(1.1)**	48.6(19.2)/ 89(2.1)
	Pronate	72.9(11.9)/93.1(1.6)	80.9(5.9)/96.3(1.3)	76.3(7.7)/94.7(1		

Note: SVM: support vector machine; RF: random forest; XGBoost: extreme gradient boosting; CNN1D: one-dimensional convolutional neuron networks; MCC: Matthews correlation coefficient. Bold fonts denote the highest accuracy for each method.

### Deep Learning Approach

The test accuracy of the foot dorsum model was 74.0 ± 3.8% for the subject-wise approach with an AUC of 0.80 ± 0.05 (Table 2). Similar performance was obtained from three sensor protocols for the record-wise method. A classification report for the CNN1D model is illustrated in Table 2.

### Data Augmentation

After adding noise, the time scale, and time warp for the training data, five-fold cross-validation test accuracy was 74.3 ± 4.2%, 72.7 ± 4.9%, and 72.3 ± 1.9%, respectively (Table 3). Accuracy was 74.1 ± 5.2% for adding noise and time warp augmentation and 72.5 ± 2.2% for adding noise and time scale.

**Table 3:** Model’s performance with different data augmentation approaches.

	Adding noise	Time scale	Time warp	Adding noise and Time warp	Adding noise and Time scale
Accuracy	74.3 ± 4.2%	72.7 ± 4.9%	72.3 ± 1.9%	74.1 ± 5.2%	72.5 ± 2.2%
MCC	47.7 ± 8.5%	48.1 ± 7.6%	46 ± 4.7%	51.4 ± 9.4	45.8 ± 4.7

Note: MCC: Matthews correlation coefficient

## Discussion

Monitoring foot posture is crucial for running injury prevention by providing runners with the right running shoes. However, there is no tool to estimate foot pronation during running. This study found that both classic feature-based machine learning algorithms and CNN1D could reliably predict foot pronation during running, specifically for the record-wise method. Sensor location significantly affected the foot pronation detection, and the sensor placed on the foot dorsum outperformed the distal tibia. The number of sensors affected feature-based algorithms (SVM and RF), but not the CNN1D model. This demonstrates that CNN1D has a higher generalization capability compared with other machine learning approaches of which performance is limited by features.

All running-related injuries are caused by an additional external or internal force generated during running. The altered loading is associated with changes in the foot shape or posture. However, the injury rate of running has not decreased noticeably over the past few decades (Malisoux et al., 2016; Nigg et al., 2015). Running posture is regarded as one of the significant running injury factors. Runners with pronated feet were more likely to sustain lower limb injuries than those with neutral feet (Malisoux et al., 2016). Neal et al. (2015) found that the pronated foot increased the incidence of medial tibia stress syndrome and patellofemoral pain.

Non-fitting and uncomfortable shoes can cause pain and further lead to running injuries (Xiang et al., 2018; Xiang et al., 2022b). Running in the right kinds of shoes is crucial to improving running performance and preventing running-related injuries. Running shoe manufacturers design different types of shoes to fit runners’ feet and tackle the potential injury risks. The well-known types are cushioning, stability, and motion control running shoes, which are recommended for runners with supinated, neutral, and pronated feet (Malisoux et al., 2016). Malisoux et al. (2016) confirmed that pronated runners wearing motion control shoes had a decreased overall injury risk compared to those wearing conventional running shoes. After fatigue induced by running, motion-control shoes can also prevent mechanical loading increases at the initial foot contact by adopting a longer shoe-ground contact time.

Classification algorithms have been previously utilized in multiple gait detection scenarios such as classifying daily activities (Ordóñez and Roggen, 2016), running surfaces (Dixon et al., 2019), and runners’ competition levels (Liu et al., 2020) and have shown great success. Detecting subtle changes during the gait has been a challenging task. Dixon et al. (2019) confirmed that CNN1D could detect outdoor terrain types during running using a single inertial sensor on the tibia. Multilayer perceptron (MLP) exhibited good accuracy in classifying runners’ performance levels (Liu et al., 2020). Hu et al. (2018) found that LSTM could detect age-related differences during the gait. However, those models were not validated on the unseen data from new subjects. On the other hand, foot posture monitoring is arduous during dynamic tasks (Behling et al., 2020). The reliable method for static foot pronation check is FPI-6 (Redmond et al., 2006). However, no study has reported how to track foot posture during running.

In this study, acceleration in tandem with gyroscope data was input to the feature-based machine learning algorithms and CNN1D for the binary classification task of being pronated or not. Furthermore, we employed a custom nested k-fold cross-validation approach to train, validate, and test our model’s performance. This structure shows the advantages of making good use of all datasets for training and testing and is also easy for building the subject-wise model. Consistent with our hypothesis, the record-wise model considerably increased classifying accuracy compared with the subject-wise model. It is suggested to validate the model’s performance on unseen data from new participants to enhance the generalization of classifiers (Tan et al., 2020). However, due to lower accuracy and overfitting in the subject-wise approach, our model was more robust based on the record-wise data. This can be improved in the future for subject-specific applications with more advanced algorithms or more sample size for training. Furthermore, two sensor signals from the foot dorsum and tibia had better performance in SVM and RF, but not the XGBoost and the signal-based deep learning CNN1D model. Therefore, the tibia IMU signal may not be able to provide helpful information about foot pronation in ensemble algorithms and neural networks. Foot pronation affects foot characteristics, but tibia shock acceleration remains unchanged.

Furthermore, this study employed the time-series data augmentation technology to add more synthetic data during model training. It demonstrated that this subject-independent model had good generalizability with an increase of the training dataset. Performance of the record-wise and subject-wise models in this study was estimated through nested k-fold cross-validation to generalize these findings. Considering a relevant small dataset in the biomechanical realm, it encourages applying nested cross-validation or leave-one-out cross-validation methods for training, validating, and evaluating the model to maximize the use of data.

This work should be viewed with some limitations. Several time-series data augmentation methods were applied in this study. However, we did not utilize advanced data augmentation methods, such as generative adversarial networks, to generate more synthetic data. The data generation technique in this study was intended to test the models’ generalization, but not to augment the sample size. FPI-6 was adopted as the ground truth in this study to evaluate foot pronation as it is clinically validated and assesses foot posture from multiple dimensions. Future studies may provide a comprehensive perspective for evaluating and predicting foot pronation during running. Furthermore, runners were not included in this study if the foot was not changed to a pronated posture to avoid data imbalance during model training and mitigate fatigue effects.

## Conclusions

This study conducted a preliminary investigation into foot pronation prediction during running with multiple machine learning algorithms. XGBoost is a recommended feature-based algorithm for identifying foot pronation during running with inertial sensor data on the foot dorsum as input. These algorithms, particularly the XGBoost and CNN1D models trained on inertial sensor data collected from the foot dorsum, could be integrated into wearable devices such as a smartwatch to monitor a runner's foot pronation during prolonged running with the goal of shoe matching and reducing or preventing foot posture-related injuries.

## References

[ref1] Ahamed, N. U., Kobsar, D., Benson, L. C., Clermont, C. A., Osis, S. T., & Ferber, R. (2019). Subject-specific and group-based running pattern classification using a single wearable sensor. Journal of Biomechanics, 84, 227–233. 10.1016/j.jbiomech.2019.01.001 30670327

[ref2] Barandas, M., Folgado, D., Fernandes, L., Santos, S., Abreu, M., Bota, P., Liu, H., Schultz, T., & Gamboa, H. (2020). TSFEL: Time series feature extraction library. SoftwareX, 11, 100456. 10.16/j.softx.2020.100456

[ref3] Behling, A. V., Manz, S., von Tscharner, V., & Nigg, B. M. (2020). Pronation or foot movement — What is important. Journal of Science and Medicine in Sport, 23(4), 366–371. 10.1016/j.jsams.2019.11.002 31776068

[ref4] Clermont, C. A., Benson, L. C., Osis, S. T., Kobsar, D., & Ferber, R. (2019). Running patterns for male and female competitive and recreational runners based on accelerometer data. Journal of Sports Sciences, 37(2), 204–211. 10.1080/02640414.2018.1488518 29920155

[ref5] Dempster, J., Dutheil, F., & Ugbolue, U. C. (2021). The Prevalence of Lower Extremity Injuries in Running and Associated Risk Factors: A Systematic Review. Physical Activity and Health, 5(1), 133–145. 10.5334/paah.109

[ref6] Dixon, P. C., Schütte, K. H., Vanwanseele, B., Jacobs, J. V., Dennerlein, J. T., Schiffman, J. M., Fournier, P. A., & Hu, B. (2019). Machine learning algorithms can classify outdoor terrain types during running using accelerometry data. Gait and Posture, 74, 176–181. 10.1016/j.gaitpost.2019.09.005 31539798

[ref7] Dorschky, E., Nitschke, M., Martindale, C. F., van den Bogert, A. J., Koelewijn, A. D., & Eskofier, B. M. (2020). CNN-based estimation of sagittal plane walking and running biomechanics from measured and simulated inertial sensor data. Frontiers in Bioengineering and Biotechnology, 8, 604. 10.3389/fbioe.2020.00604 32671032 PMC7333079

[ref8] Dos Santos, J. O. L., Gomes, A. L. R., Lima, A. B., de Paiva Vieira, E., Bezerra, E.., de S., & Rossato, M. (2019). Effect of linear running velocity on the increase on foot pronation. Foot, 41, 74–78. 10.1016/j.foot.2019.09.004 31733448

[ref9] Halilaj, E., Rajagopal, A., Fiterau, M., Hicks, J. L., Hastie, T. J., & Delp, S. L. (2018). Machine learning in human movement biomechanics: Best practices, common pitfalls, and new opportunities. Journal of Biomechanics, 81, 1–11. 10.1016/j.jbiomech.2018.09.009 30279002 PMC6879187

[ref10] Hernandez, V., Dadkhah, D., Babakeshizadeh, V., & Kulić, D. (2021). Lower body kinematics estimation from wearable sensors for walking and running: A deep learning approach. Gait and Posture, 83, 185–193. 10.1016/j.gaitpost.2020.10.026 33161275

[ref11] Hu, B., Dixon, P. C., Jacobs, J. V., Dennerlein, J. T., & Schiffman, J. M. (2018). Machine learning algorithms based on signals from a single wearable inertial sensor can detect surface-and age-related differences in walking. Journal of Biomechanics, 71, 37–42. 10.1016/j.jbiomech.2018.01.005 29452755

[ref12] Kettaneh, N., Berglund, A., & Wold, S. (2005). PCA and PLS with very large data sets. Computational Statistics & Data Analysis, 48(1), 69–85. 10.1016/j.csda.2003.11.027

[ref13] Kobsar, D., Osis, S. T., Hettinga, B. A., & Ferber, R. (2014). Classification accuracy of a single tri-axial accelerometer for training background and experience level in runners. Journal of Biomechanics, 47(10), 2508–2511. 10.1016/j.jbiomech.2014.04.017 24837221

[ref14] Liu, Q., Mo, S., Cheung, V. C. K., Cheung, B. M. F., Wang, S., Chan, P. P. K., Malhotra, A., Cheung, R. T. H., & Chan, R. H. M. (2020). Classification of runners’ performance levels with concurrent prediction of biomechanical parameters using data from inertial measurement units. Journal of Biomechanics, 112, 110072. 10.1016/j.jbiomech.2020.110072 33075666

[ref15] Malisoux, L., Chambon, N., Delattre, N., Gueguen, N., Urhausen, A., & Theisen, D. (2016). Injury risk in runners using standard or motion control shoes: A randomised controlled trial with participant and assessor blinding. British Journal of Sports Medicine, 50(8), 481–487. 10.1136/bjsports-2015-095031 26746907 PMC4853529

[ref16] Mei, Q., Gu, Y., Xiang, L., Baker, J. S., & Fernandez, J. (2019). Foot pronation contributes to altered lower extremity loading after long distance running. Frontiers in Physiology, 10, 573. 10.3389/fphys.2019.00573 31191329 PMC6540596

[ref17] Neal, B. S., Griffiths, I. B., Dowling, G. J., Murley, G. S., Munteanu, S. E., Franettovich Smith, M. M., Collins, N. J., & Barton, C. J. (2015). Foot posture as a risk factor for lower limb overuse injury: A systematic review and meta-analysis. Journal of Foot and Ankle Research, 7, 55. 10.1186/s13047-014-0053-6 PMC4282737 25558288

[ref18] Ngoh, K. J.-H., Gouwanda, D., Gopalai, A. A., & Zheng, C. Y. (2018). Estimation of vertical ground reaction force during running using neural network model and uniaxial accelerometer. Journal of Biomechanics, 76, 269–273. 10.1016/j.jbiomech.2018.06.006 29945786

[ref19] Nigg, B., Behling, A., & Hamill, J. (2019). Foot pronation. Footwear Science, 11(3), 131–134. 10.1080/19424280.2019.1673489

[ref20] Nigg, B. M., Baltich, J., Hoerzer, S., & Enders, H. (2015). Running shoes and running injuries: mythbusting and a proposal for two new paradigms:‘preferred movement path’and ‘comfort filter.’ *British Journal of Sports Medicine*, 49(20), 1290–1294. 26221015 10.1136/bjsports-2015-095054

[ref21] Ordóñez, F. J., & Roggen, D. (2016). Deep convolutional and LSTM recurrent neural networks for multimodal wearable activity recognition. Sensors, 16, 115. 10.3390/s16010115 26797612 PMC4732148

[ref22] Pan, J. W., Ho, M. Y. M., Loh, R. B. C., Iskandar, M. N. S., & Kong, P. W. (2023). Foot Morphology and Running Gait Pattern between the Left and Right Limbs in Recreational Runners. Physical Activity and Health, 7(1), 43–52. 10.5334/paah.226

[ref23] Redmond, A. C., Crane, Y. Z., & Menz, H. B. (2008). Normative values for the Foot Posture Index. Journal of Foot and Ankle Research, 1(1), 6. 10.1186/1757-1146-1-6 18822155 PMC2553778

[ref24] Redmond, A. C., Crosbie, J., & Ouvrier, R. A. (2006). Development and validation of a novel rating system for scoring standing foot posture: The Foot Posture Index. Clinical Biomechanics, 21(1), 89–98. 10.1016/j.clinbiomech.2005.08.002 16182419

[ref25] Ryan, M., Elashi, M., Newsham-West, R., & Taunton, J. (2014). Examining injury risk and pain perception in runners using minimalist footwear. British Journal of Sports Medicine, 48(16), 1257–1262. 10.1136/bjsports-2012-092061 24357642

[ref26] Saragiotto, B. T., Yamato, T. P., Hespanhol Junior, L. C., Rainbow, M. J., Davis, I. S., & Lopes, A. D. (2014). What are the main risk factors for running-related injuries? Sports Medicine, 44(8), 1153–1163. 10.1007/s40279-014-0194-6 24809248

[ref27] Tan, T., Strout, Z. A., & Shull, P. B. (2020). Accurate impact loading rate estimation during running via a subject-independent convolutional neural network model and optimal IMU placement. IEEE Journal of Biomedical and Health Informatics, 25(4), 1215–1222. 10.1109/jbhi.2020.3014963 32763858

[ref28] Um, T. T., Pfister, F. M. J., Pichler, D., Endo, S., Lang, M., Hirche, S., Fietzek, U., & Kulić, D. (2017). Data augmentation of wearable sensor data for parkinson’s disease monitoring using convolutional neural networks. Proceedings of the 19th ACM International Conference on Multimodal Interaction, 216–220.

[ref29] Xiang, L., Mei, Q., Fernandez, J., & Gu, Y. (2018). Minimalist shoes running intervention can alter the plantar loading distribution and deformation of hallux valgus: A pilot study. Gait and Posture, 65, 65–71. 10.1016/j.gaitpost.2018.07.002 30558948

[ref30] Xiang, L., Wang, A., Gu, Y., Zhao, L., Shim, V., & Fernandez, J. (2022a). Recent machine learning progress in lower limb running biomechanics with wearable technology: A systematic review. Frontiers in Neurorobotics, 16, 913052. 10.3389/fnbot.2022.913052 35721274 PMC9201717

[ref31] Xiang, L., Mei, Q., Wang, A., Shim, V., Fernandez, J., & Gu, Y. (2022b). Evaluating function in the hallux valgus foot following a 12-week minimalist footwear intervention: A pilot computational analysis. Journal of Biomechanics, 132, 110941. 10.1016/j.jbiomech.2022.110941 35063832

[ref32] Zrenner, M., Gradl, S., Jensen, U., Ullrich, M., & Eskofier, B. M. (2018). Comparison of different algorithms for calculating velocity and stride length in running using inertial measurement units. Sensors, 18(12), 4194. 10.3390/s18124194 30513595 PMC6308955

